# Acute onset lactobacillus endophthalmitis after trabeculectomy: a case report

**DOI:** 10.1186/1752-1947-4-203

**Published:** 2010-06-30

**Authors:** Dimitris Papaconstantinou, Ilias Georgalas, Themis Karmiris, Ioannis Ladas, Konstantinos Droutsas, Gerasimos Georgopoulos

**Affiliations:** 1 Department of Ophthalmology, "G. Gennimatas" Hospital, University of Athens, Greece

## Abstract

**Introduction:**

We report a case of early lactobacillus endophthalmitis which occurred ten days after trabeculectomy.

**Case presentation:**

A 76-year-old Caucasian diabetic woman underwent uncomplicated trabeculectomy with a collagen implant as an adjunct, in her left phakic eye, for the treatment of uncontrolled open-angle glaucoma. Ten days post-operatively, our patient complained of left phakic eye discharge pain and visual acuity decreased to "light-perception". The anterior chamber had 3+ cells and flare, and there was also 2 mm layered hypopyon. Vitreous involvement was present obscuring visualization of the fundus. On the same day our patient underwent vitrectomy surgery and intra-vitreal and systemic antibiotics were administered. Vitreous cultures grew *Lactobacillus brevis*. Our patient responded well to treatment and 30 days after vitrectomy visual acuity improved to 1/10. Six months later our patient underwent cataract surgery. Eight months after initial surgery visual acuity was 2/10 and intra-ocular pressure was 14 mmHg without any anti-glaucoma medication.

**Conclusions:**

This is the first report of acute lactobacillus endophthalmitis in the phakic eye of a diabetic patient after trabeculectomy. Glaucoma surgeons should be aware of the potential for acute post-operative endophthalmitis due to rare microorganisms, such as lactobacillus, in glaucoma filtration surgery, especially in diabetic patients. The literature shows an increased risk of endophthalmitis when anti-metabolites are used in conjunction with trabeculectomy. Perhaps, any type of wound healing modulation, such as collagen or mitomycin-C may increase this risk. However, it is unclear at this time and more studies need to be done. In this single case, vitrectomy combined with intra-vitreal and systemic antibiotics were efficient in limiting the devastating sequels of this complication.

## Introduction

Trabeculectomy is the most common operation for the treatment of glaucoma worldwide. However, bleb failure may be eventually induced, by wound healing processes and scar formation [1,2]; many adjunctive procedures such as the use of anti-metabolites [3], bleb needling and implants have been proposed to enhance and maintain the hypotensive effect of trabeculectomy [4,5]. Ologen™ is a bioengineered, biodegradable soaked, three-dimensional, porous, collagen-glycosaminglycan implant which may be used in trabeculectomy, providing controlled resistance between the anterior chamber and the subconjunctival space [4,6]. In a recent study trabeculectomy with Ologen has not been proven to offer any significant advantages when compared with trabeculectomy alone [6]. Additionally, one of the studied eyes developed endophthalmitis but this was not clearly associated with the Ologen use [6].

Lactobacillus is a gram-positive microorganism that is generally considered non-pathogenic and it is used for enhancement of flavor in dairy products. We report a case of early endophthalmitis due to *Lactobacillus brevis *after trabeculectomy, which, to the best of our knowledge, has never been reported before as a cause for post-surgical endophthalmitis, and describe its course after vitrectomy surgery.

## Case presentation

A 76-year-old Caucasian woman was admitted for a trabeculectomy in the left phakic eye (LE) for uncontrolled open-angle glaucoma. Visual acuity (VA) was 4/10. The patient denied a history of systemic illness apart from type II diabetes mellitus.

**Figure 1:**
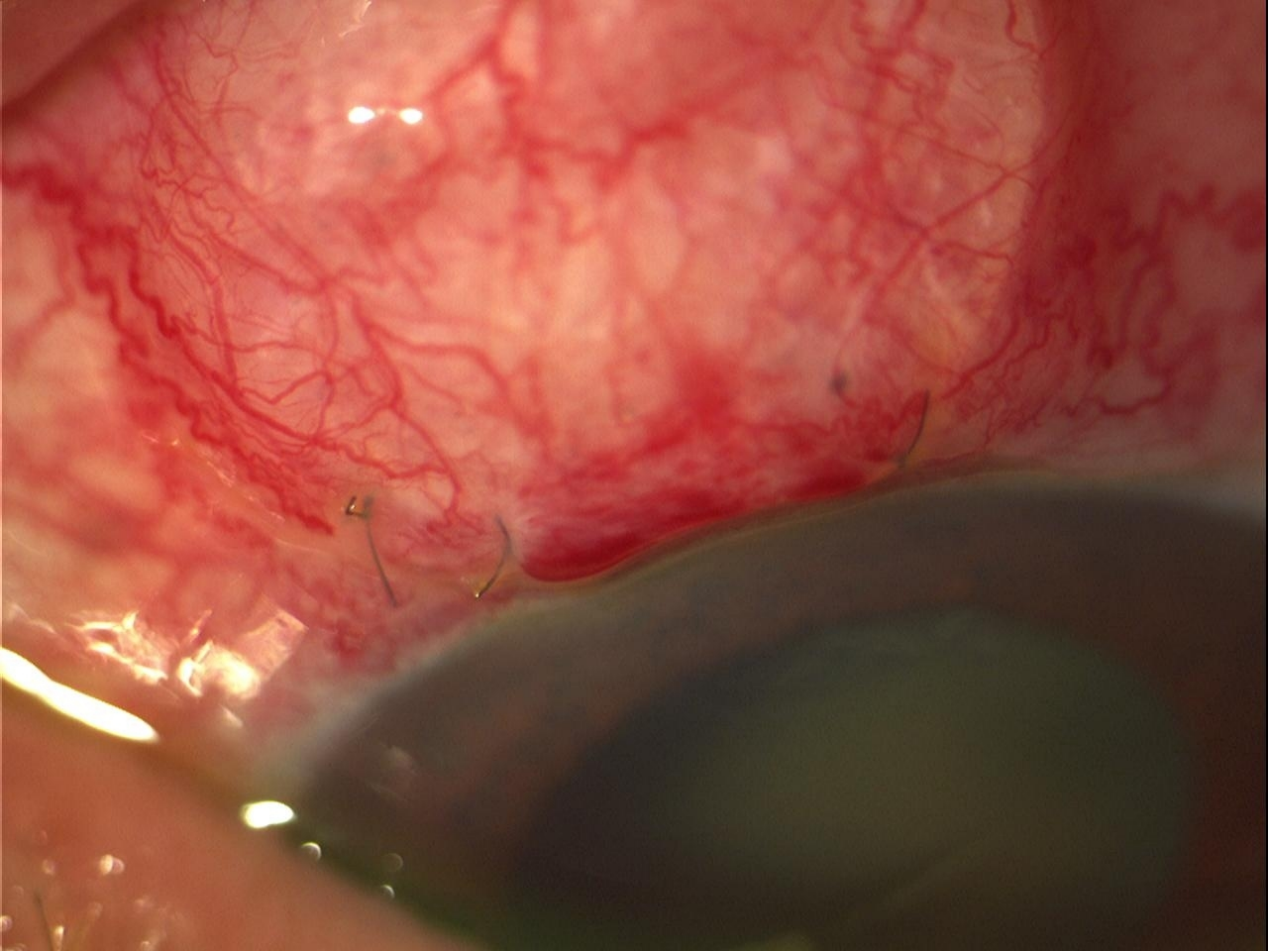
**First day after trabeculectomy with Ologen, the anterior chamber is well formed and the Ologen implant *in situ***.

**Figure 2:**
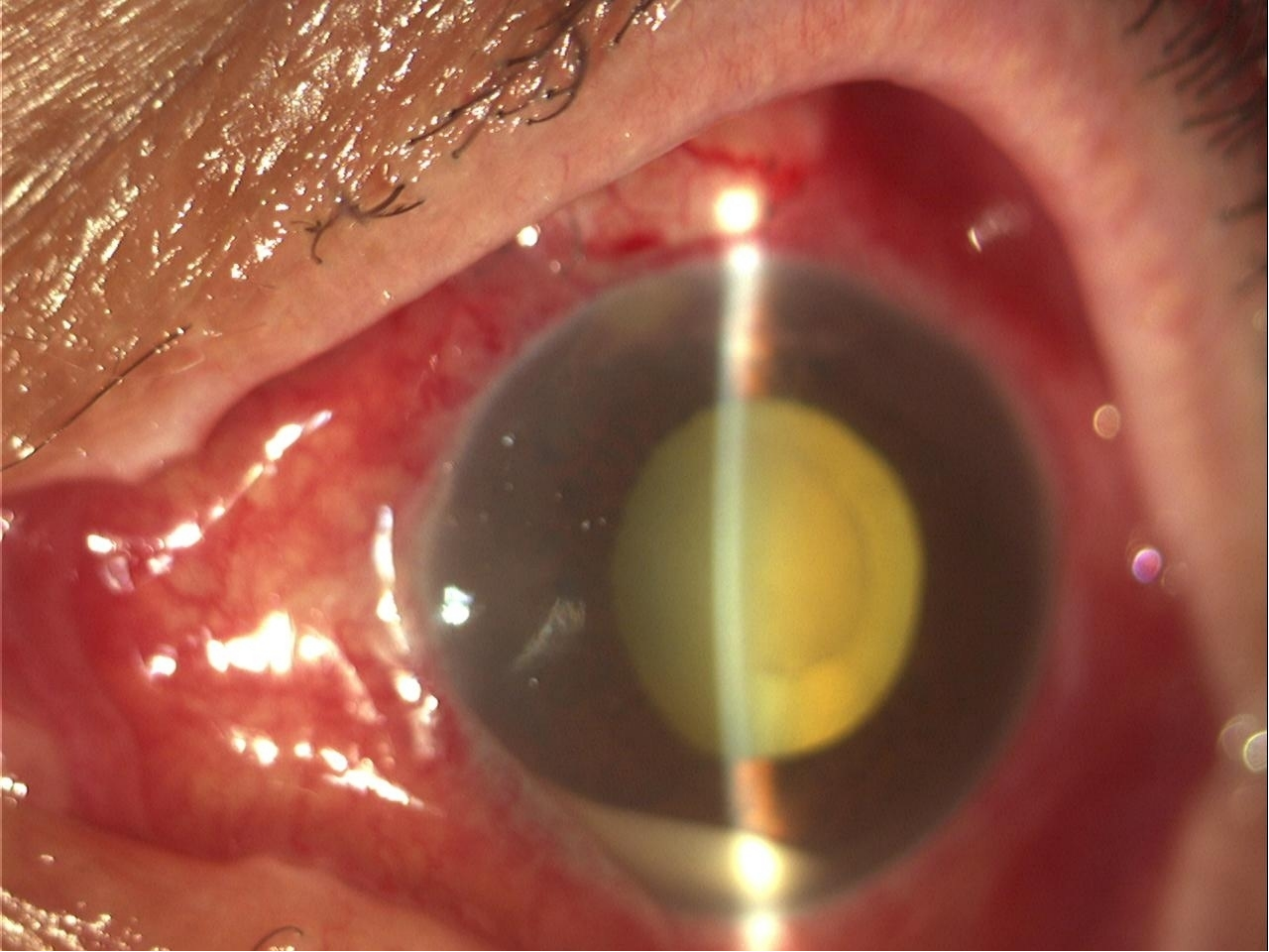
**Ten days after trabeculectomy, there is hypopyon with plastic fibrin across the papillary margin**.

Uncomplicated trabeculectomy was performed during which an Ologen implant was placed on top of the scleral flap under the conjunctiva before the final suturing. On the first post-operative day, there was a diffuse superior bleb. The anterior chamber (AC) was half normal depth and contained a minimal reaction of 1+ cells and flare (Figure 1). One week post-operatively the situation was unchanged. Ten days post-operatively, our patient complained of LE discharge pain and VA decreased to "light-perception". Anterior chamber had 3+ cells and flare, and there was also 2 mm layered hypopyon with plastic fibrin across the pupillary margin (Figure 2). Vitreous involvement was present obscuring visualization of the fundus.

On the same day the patient underwent vitrectomy, during which the vitreous cutter was placed in the mid vitreous and before turning on the infusion, 0.5 mL vitreous sample was aspirated. Subsequently the infusion was turned on and the cortical vitreous was excised. An intra-vitreal injection of vancomycin and amikacin was administered. The Ologen implant was not removed from the trabeculectomy since there were no signs of severe blebitis. Vitrectomy cassette and samples were sent to the laboratory. Ologen implants from the same batch, with the one used, were sent for culture; additionally, blood samples and samples from our patient's conjunctiva were send for culture. Avelox tablets 400 mg (moxifloxacin-hydrochloride) twice a day, ofloxacin and dexamethasone drops four times a day and atropine drops twice a day, were administered post-operatively.

**Figure 3:**
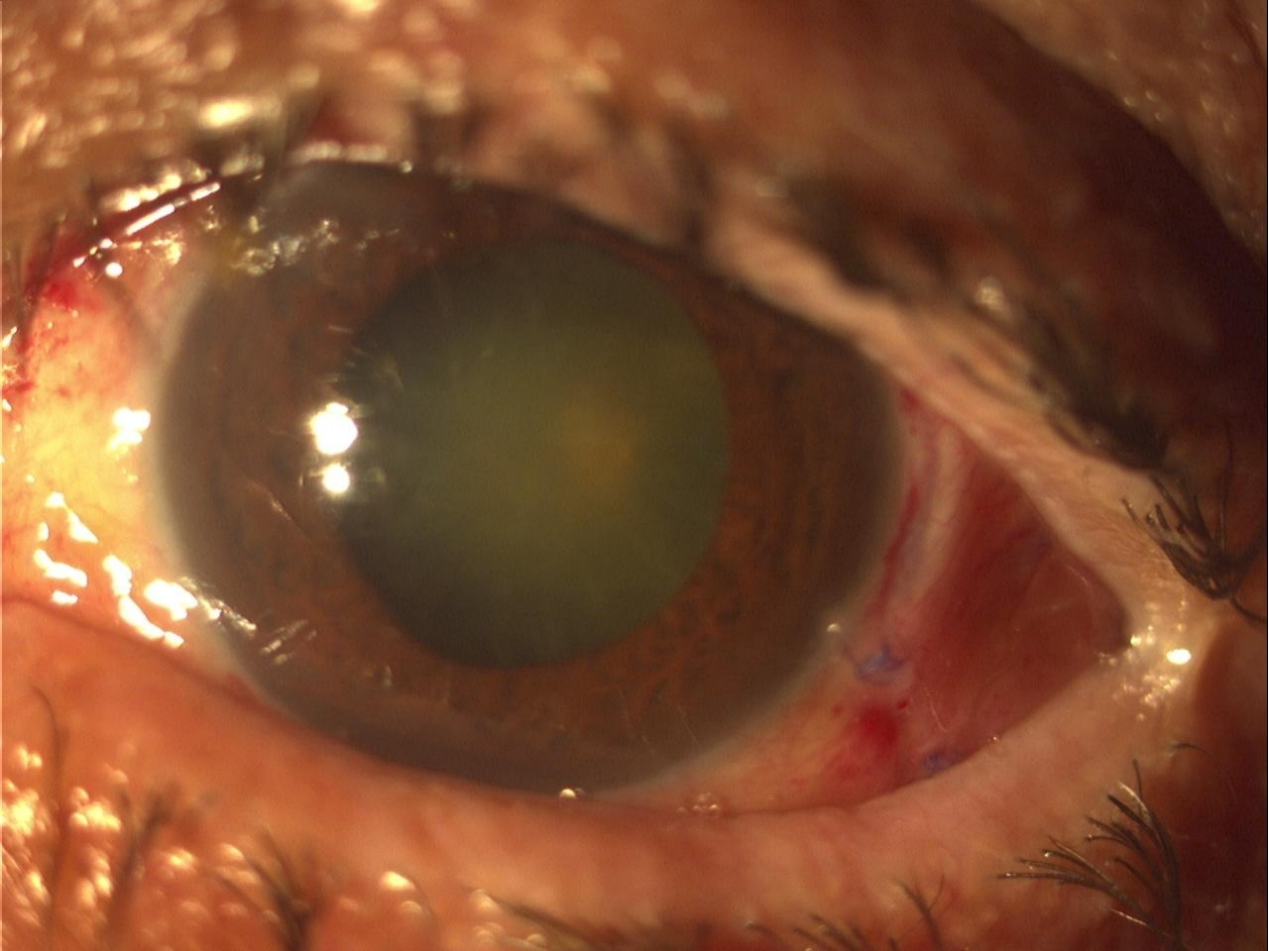
After vitrectomy hypopyon has completely subsided.

Our patient responded well to treatment and infection started resolving from the first post-operative day. Cultures from both undiluted vitreous and cassette grew *Lactobacillus brevis*, blood and cultures from the conjunctival samples and the collagen implants (from the same batch) were negative for any microorganism. One week later fundus reflex was visible in slit-lamp biomicroscopy (Figure 3), and 30 days later VA was 1/10 and intra-ocular pressure 14 mmHg. Six months later our patient underwent uncomplicated phacoemulsification and intra-ocular lens implantation. Eight months after initial surgery VA is 2/10 and intra-ocular pressure is 14 mmHg without any anti-glaucoma medication.

## Discussion

Bacterial endophthalmitis, after trabeculectomy, usually develops months to years after surgery. Acute post-operative endophthalmitis has been rarely reported in phakic eyes after trabeculectomy [7]. Eifrig *et al*. in a review of the medical records of 35,916 patients that underwent intra-ocular surgery, found only four patients who developed acute endophthalmitis after trabeculectomy, in three of whom mitomycin-C (MMC) had been used as an adjunct [8]. Kuang *et al*. [9] in a retrospective study reported only one case, out of 988 trabeculectomies, with early post-operative endophthalmitis due to *Morganella morganii*. Host flora is usually responsible for early cases of endophthalmitis and *Staphylococcus epidermitis *is the most common organism. Gram-negative organisms such as *H. influenzae *are isolated most frequently from late onset endophthalmitis [7,8]. Most literature suggests that patients with diabetes are at increased risk of developing endophthalmitis after intraocular surgery [10].

Lactobacillus is a gram-positive bacterium and is a common inhabitant of the human mouth, gastrointestinal tract, and female genital tract. Only 200 cases of lactobacillus-associated infections have been reported in the literature [11] and only one case of endophthalmitis due to lactobacillus has been reported which concerned and injured eye [11,12]. In our case no anti-metabolite was used, but an Ologen implant was placed on top of the scleral flap under the conjunctiva, before the final suturing of the conjunctiva. The endophthalmitis occurred 10 days after surgery and we decided to perform vitrectomy first since endophthalmitis after trabeculectomy is usually severe and aggressive treatment, with vitrectomy and systemic antibiotics is advocated [13]; and second in order to yield enough undiluted vitreous for culture.

## Conclusions

We report the first case of acute lactobacillus endophthalmitis in the phakic eye of a diabetic patient after trabeculectomy. Glaucoma surgeons should be aware of the potential of acute post-operative endophthalmitis due to rare microorganisms, such as lactobacillus, in glaucoma filtration surgery, especially in diabetic patients. The literature shows an increased risk of endophthalmitis when anti-metabolites are used in conjunction with trabeculectomy. Perhaps any type of wound healing modulation, such as collagen or mitomycin-C, may increase this risk; however, it is unclear at this time and more studies needs to be done. In this case, vitrectomy combined with intra-vitreal and systemic antibiotics were efficient in limiting the devastating sequels of this complication.

## Consent

Written informed consent was obtained from the patient for publication of this case report and any accompanying images. A copy of the written consent is available for review by the Editor-in-Chief of this journal.

## Competing interests

The authors declare that they have no competing interests.

## Authors' contributions

DP, IG and IL were in charge of the medical care of the patient and performed the different surgeries. KD, TK were responsible for the photographs and the literature review. DP, IG and KD wrote the manuscript. GG and IL reviewed it, drafted it critically and provided helpful comments. All authors read and approved the final manuscript.
